# Unveiling the Connections Between Melanoma Differentiation-Associated Gene 5 (MDA5)-Positive Dermatomyositis and Its Potential Association With COVID-19: A Report of Two Cases

**DOI:** 10.7759/cureus.65016

**Published:** 2024-07-20

**Authors:** Temitope B Abegunde, Christophe Persad, Sojibuchi Ojule, Jane Colgan, Margaret Duncan

**Affiliations:** 1 Internal Medicine, University Hospital Ayr, Ayr, GBR; 2 Rheumatology, University Hospital Ayr, Ayr, GBR; 3 Internal Medicine, University Hospital Crosshouse, Kilmarnock, GBR; 4 Dermatology, University Hospital Crosshouse, Kilmarnock, GBR

**Keywords:** case report, sars-cov-2, covid-19, dermatomyositis, anti-mda5 autoantibody

## Abstract

Dermatomyositis is a rare inflammatory condition affecting the skin, muscles, and joints. This series of anti-melanoma differentiation-associated gene 5 (anti-MDA5) autoantibody-positive cases highlights a possible link between anti-MDA5-positive dermatomyositis and COVID-19 exposure. A retrospective analysis was performed on anti-MDA5-positive dermatomyositis patients at a district general hospital between January 2021 and July 2023. Information was gathered on the clinical profiles, diagnostics, management, and disease course. The two cases are as follows: (1) A 44-year-old Asian female presented with back pain, tender proximal muscles, symmetrical synovitis, and Gottron’s papules, which gradually began after a COVID vaccine and worsened after COVID-19 infection. Despite prompt management, she required finger amputation and a switch to immunomodulators to achieve arthritic disease control. (2) A 49-year-old Caucasian female presented with progressive dyspnea, polyarthralgia, dusky maculopapular rash, and oral ulcers, which began after a COVID vaccine and worsened after COVID-19 infection. Steroids and immunomodulators improved mobility and respiratory symptoms, while biologics subdued her skin symptoms. This case study provides growing evidence for an intriguing association between COVID-19 exposure and anti-MDA5 antibody-positive dermatomyositis. Further research is required to elucidate the underlying pathogenic mechanism. Early involvement of a multidisciplinary team, consideration of symptom variety, infection vigilance, and impact on quality of life are important factors for clinicians to consider when tailoring the management of these patients for optimized outcomes.

## Introduction

Dermatomyositis (DM) is a rare inflammatory condition that affects the skin, muscles, and joints. Its prevalence was calculated to be 0.00013% in the European and the United States of America (USA) population. The incidence in the United Kingdom (UK) and Ireland was calculated to be 1.9/million/year (95% CI: 1.4,2.6) [1]. There are several myositis-specific autoantibodies (MSA), each with its own clinical manifestations. The common MSAs include anti-Mi-2, anti-TIF-1-γ (anti-transcriptional intermediary factor 1 γ), anti-NXP2 (anti-nuclear matrix protein 2), anti-SAE (anti-small ubiquitin-like modifier-1 activating enzyme), and anti-MDA5 (anti-melanoma differentiation-associated gene 5) antibodies [2]. Of these, only 7%-13% of cases show anti-MDA5 positivity [3]. This unique subset of dermatomyositis can present with the typical skin manifestations of DM, including periorbital heliotrope rash; erythematous rash on the chest (V-sign), back, and shoulders (Shawl sign), and Gottron’s papules. It also has several features that distinguish it from the classical subtype, including rapidly progressive interstitial lung disease (RP-ILD) and amyopathic or hypomyopathic presentation [3,4]. Anti-MDA5 DM is also particularly associated with palmar papules and skin ulcers, often occurring in Gottron’s papules [5,6]. Three phenotypes have been suggested [3,4]. The first is associated with arthralgia/arthritis and minimal ILD [3,4]. The second involves skin vasculopathy and proximal muscle weakness; unlike the other two, it is more common in men [3,4]. Finally, the third phenotype has the highest mortality rate and is associated with RP-ILD and mechanical hands, a sign also commonly seen in anti-synthetase syndrome [3,4].

Because of its rarity, a high-quality randomized control trial (RCT) is yet to be completed [6]. Therefore, no standard treatment is available, and case studies are used as references to formulate management plans [6]. This series of anti-MDA5 autoantibody-positive cases found at University Hospital Ayr in Ayrshire, Scotland, highlights the possible link between anti-MDA5-positive dermatomyositis and COVID-19 exposure.

This case report was previously presented as an oral presentation at the In2Derm conference hosted by the Scottish Dermatology Society (SDS) on October 5, 2023. It was also presented as a poster at the Scottish Society of Rheumatology (SSR) Annual Autumn Meeting on November 2-3, 2023, and was published as an abstract on their website thereafter.

## Case presentation

Case 1

**Figure 1:**
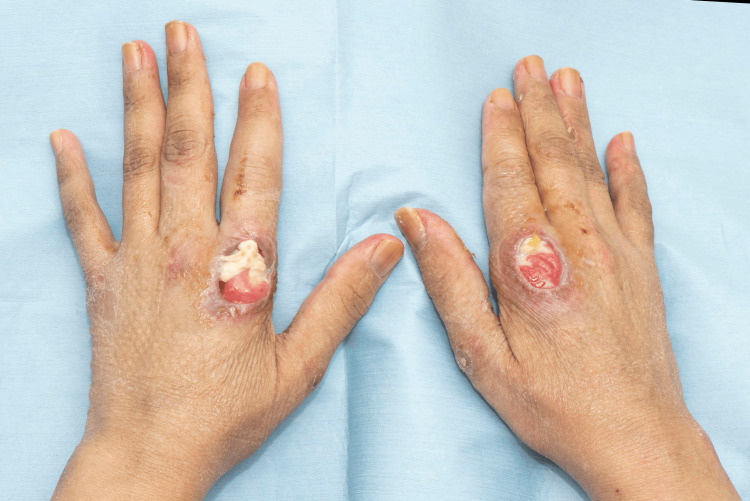
Ulcerated Gottron's papule on the MCP joints of the index fingers of patient 1 MCP: Metacarpophalangeal.

**Figure 2:**
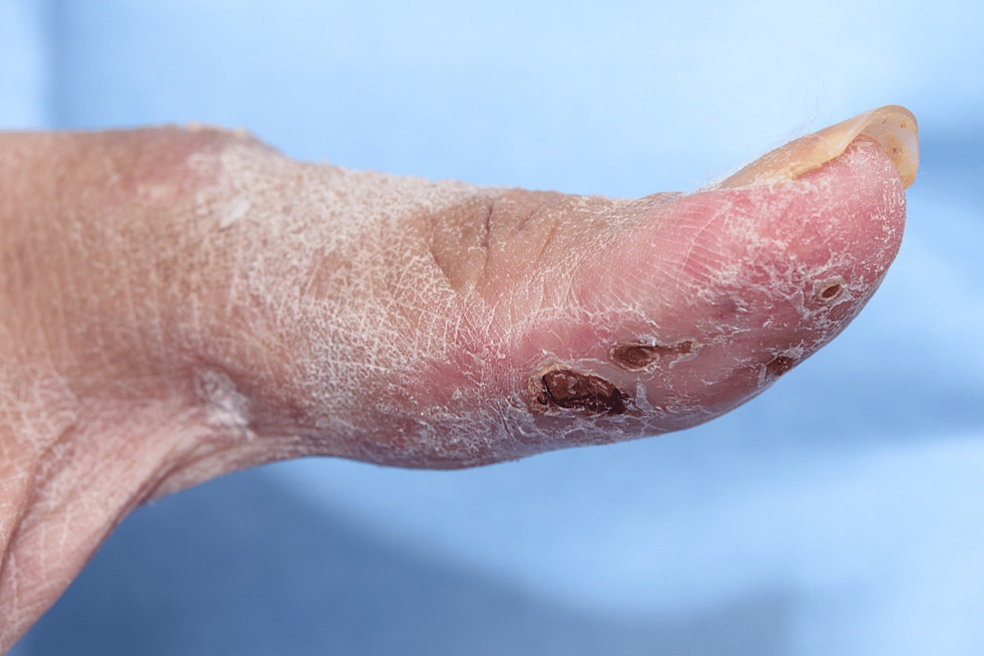
Mechanic's hand lesion with a few crusted erosions on the right thumb of patient 1

A 44-year-old otherwise healthy Asian female presented with a history of back pain, which gradually began after the Pfizer COVID-19 vaccination. Four months after the post-COVID-19 infection, the patient developed an angioedematous face and a widespread maculopapular rash. There were also clinical signs of arthritis, Gottron’s papules (Figure 1), Holster sign, heliotrope rash, mechanic's hand (Figure 2), mouth ulcerations, voice hoarseness, hair thinning, and paraesthesia of her arms. Investigations revealed elevated levels of creatinine kinase and ferritin. The autoantibody panel results revealed anti-MDA5 autoantibody positivity.

**Figure 3:**
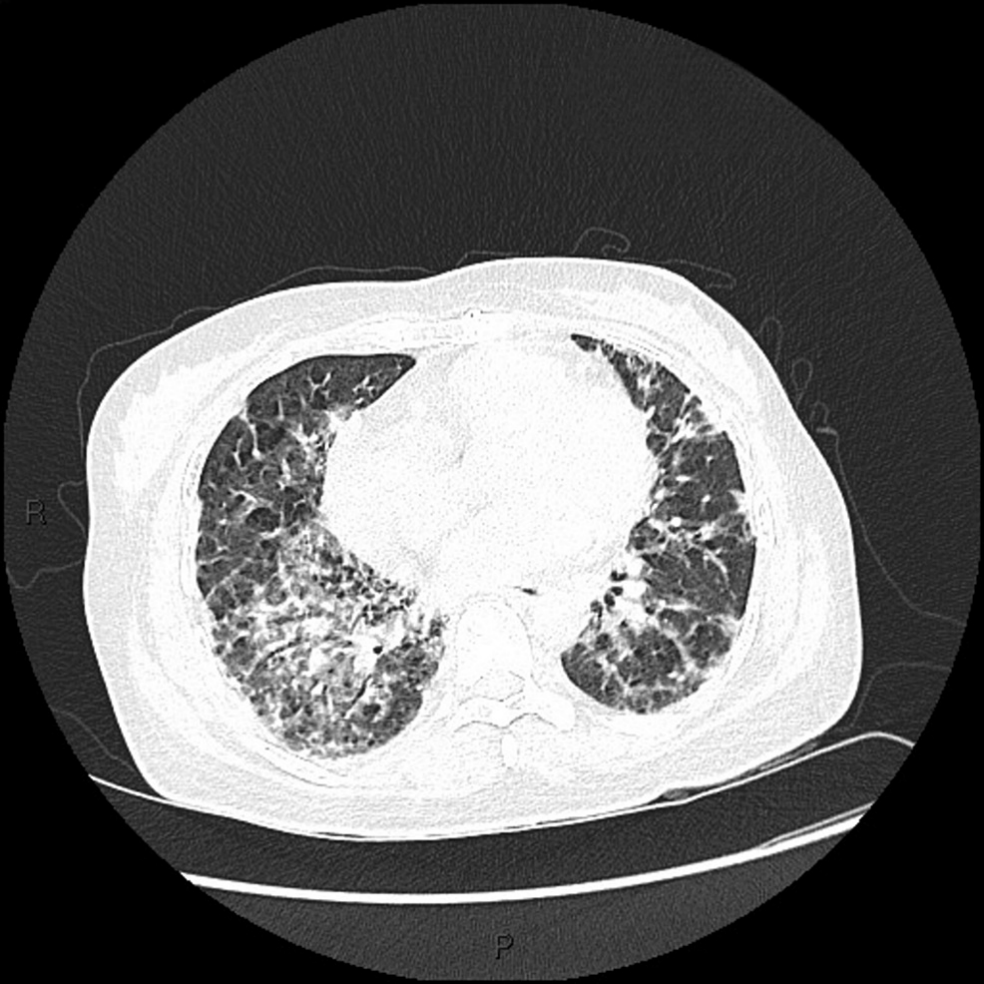
HRCT of patient 1 showing ground grass opacities in bilateral lung bases HRCT: High-resolution computed tomography.

**Figure 4:**
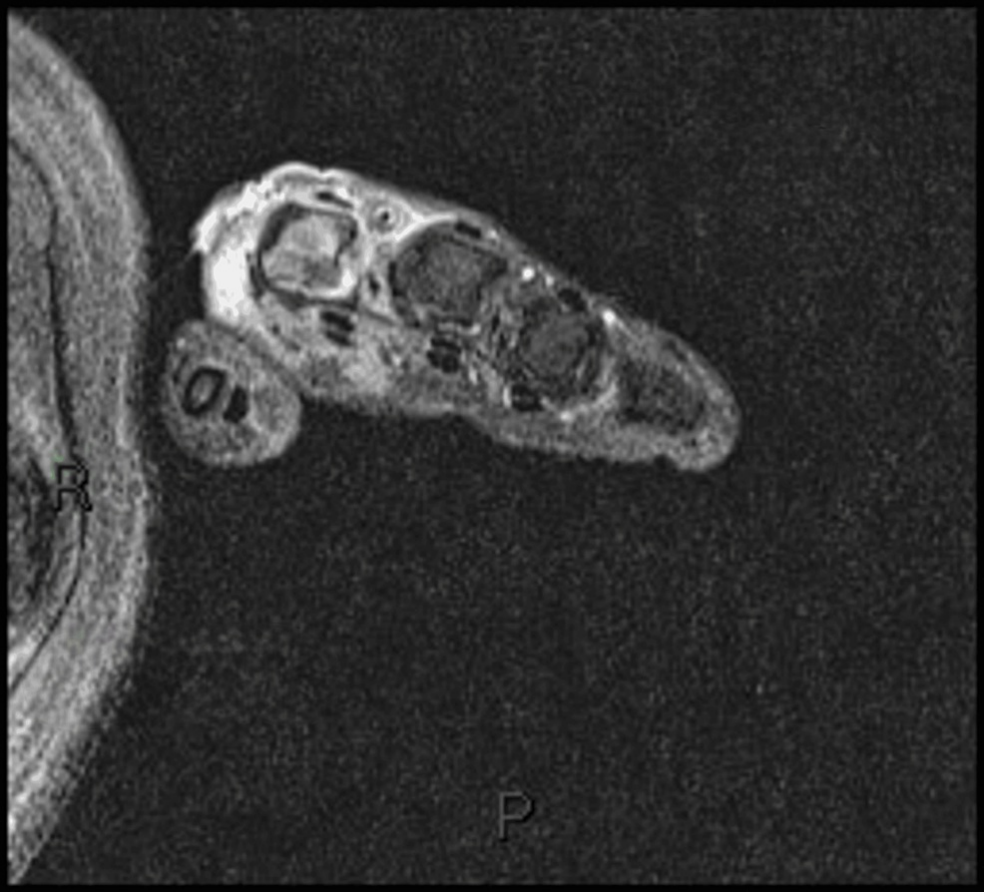
MRI of patient 1 showing inflammatory changes to the right MCP joint of the index finger with associated muscle damage MRI: Magnetic resonance imaging; MCP: Metacarpophalangeal.

Ground-glass opacities were observed on chest high-resolution computed tomography (HRCT) (Figure 3), and inflammatory muscle damage was noted on magnetic resonance imaging (MRI) (Figure 4). The patient was diagnosed with anti-MDA5 antibody-positive DM.

She was immediately started on oral prednisolone (40 mg), zoledronic acid, calcium, cholecalciferol, and omeprazole. The 1.5-g mycophenolate showed poor results after four months of treatment and worsened hair loss. It was then replaced with six cycles of cyclophosphamide, successfully gaining control over arthritic disease progression. Azathioprine, titrated up to 100 mg over a year, was next tested and produced satisfactory results in myositis suppression despite her low thiopurine methyltransferase (TPMT) enzyme status. Intravenous immunoglobulin (IVIG) was also given. Her skin symptoms were treated with topical fluocinolone 0.025% gel and hydrocortisone 1% ointment and then switched to tacrolimus 0.1% with sun protection. She developed osteoporosis from long-term steroid use after the first year. She was then switched to alendronic acid and developed esophageal perforation as a side effect, which was managed conservatively. Despite prompt management, her Gottron's papule progressed and ulcerated. The head of the metacarpophalangeal (MCP) joint became visible and necrotic, requiring finger amputation for a necrotized second MCP joint in the left hand. She was prescribed a two-year-long tapering dose of prednisolone with a maintenance dose of 5 mg. Azathioprine treatment and prophylactic co-trimoxazole 960 mg thrice weekly were continued. Her most recent HRCT showed improvement in pulmonary fibrosis. After two years, the patient developed hyperpigmentation on her forehead and erythema on the rest of her face. Her hair has now regrown. Her recent bone densitometry scan has shown a reversal of the bone mineral density loss.

Case 2

**Figure 5:**
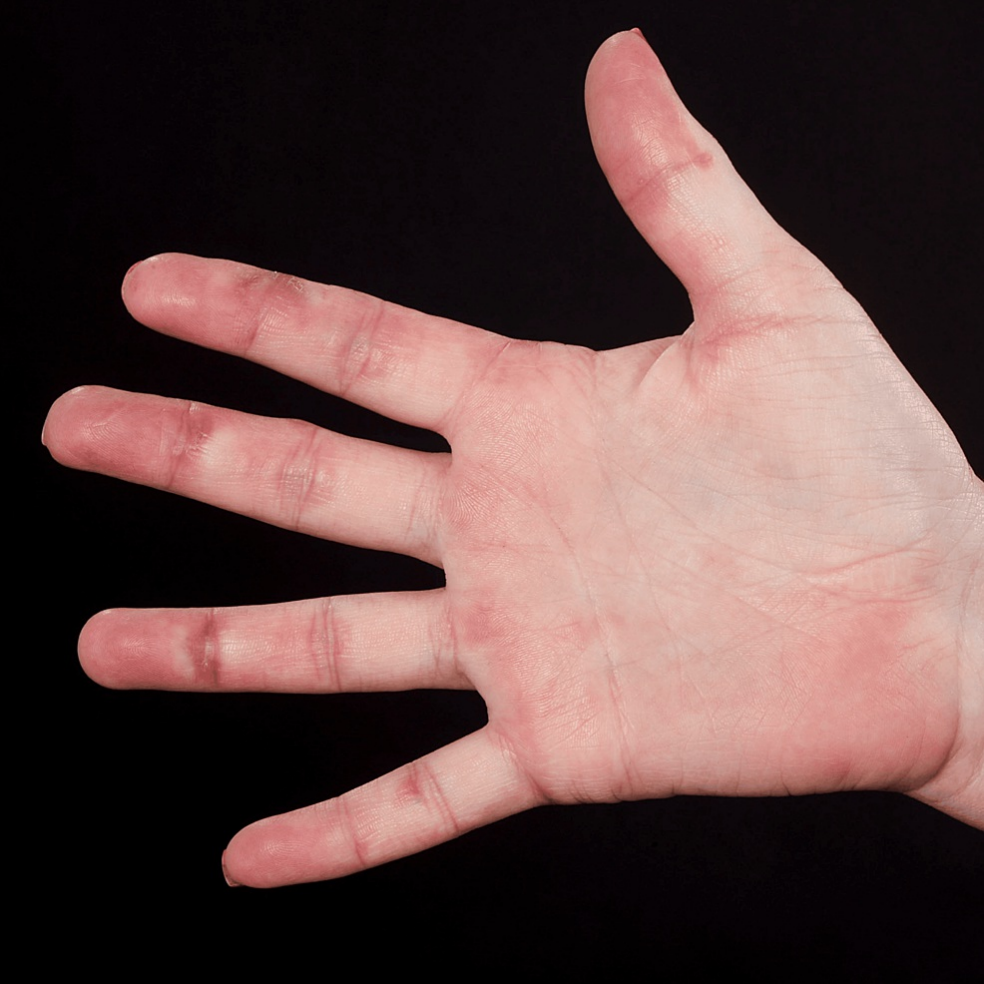
Palmar papules on the distal end of fingers on the right hand of patient 2

**Figure 6:**
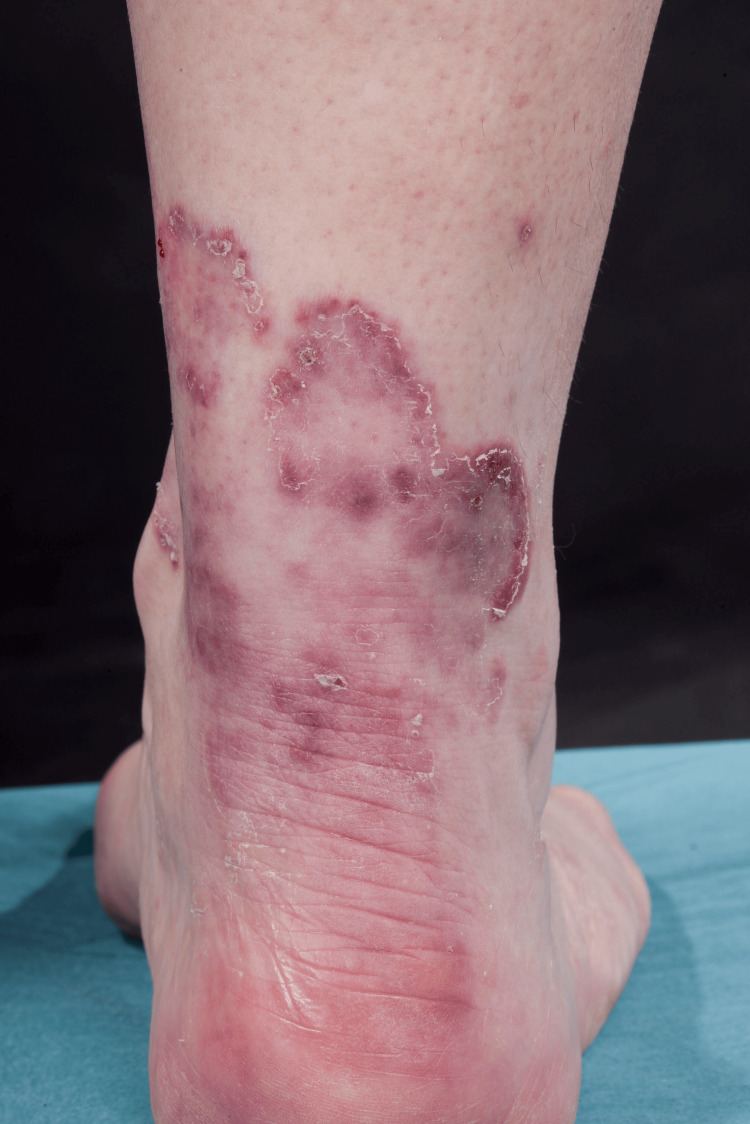
Multiple arcuate erythematous plaques with marginal scaling on the right lower leg of patient 2

A 49-year-old otherwise healthy Caucasian female presented with progressive dyspnea, polyarthralgia, and palpitations, which began after the Pfizer COVID-19 vaccination. One month after the COVID-19 infection, a dusky maculopapular facial rash and mucocutaneous ulcers developed. The preliminary diagnosis was undifferentiated connective tissue disease with differentials of post-COVID reactive arthritis and seronegative arthritis. In the following months, she developed palmar papules (Figure 5) and multiple plaque-like erythematous/purple lesions on her hips, knees, and lower legs (Figure 6).

**Figure 7:**
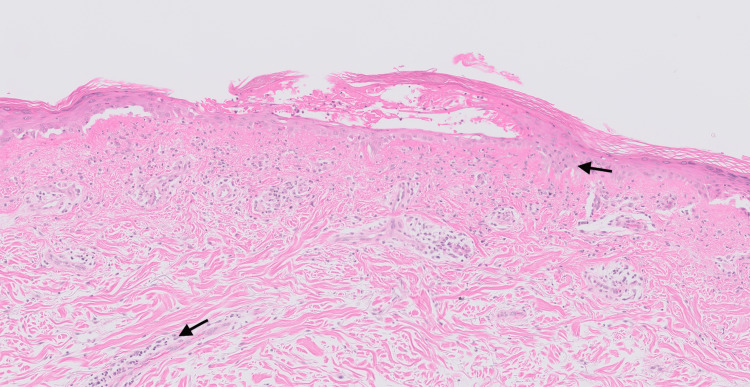
Histopathology slide of the rash on the upper back of patient 2 This shows vacuolar degeneration of the basal cell layer associated with the presence of lymphocyte infiltrates (black arrows) along the dermal-epidermal junction, hair follicles, and other appendages.

On capillaroscopy, scleroderma pattern vascular nail fold changes were observed. Investigations revealed normal creatinine kinase and elevated ferritin levels. An autoantibody panel revealed anti-SSA/Ro and anti-MDA5 antibodies. Skin biopsy showed non-specific chronic inflammatory cells suggestive of cutaneous lupus (Figure 7). The biopsy samples were negative for fungal organisms on microscopy. However, skin scrapings were positive for dermatophytes suggesting super-imposed fungal infection (Figure 6).

**Figure 8:**
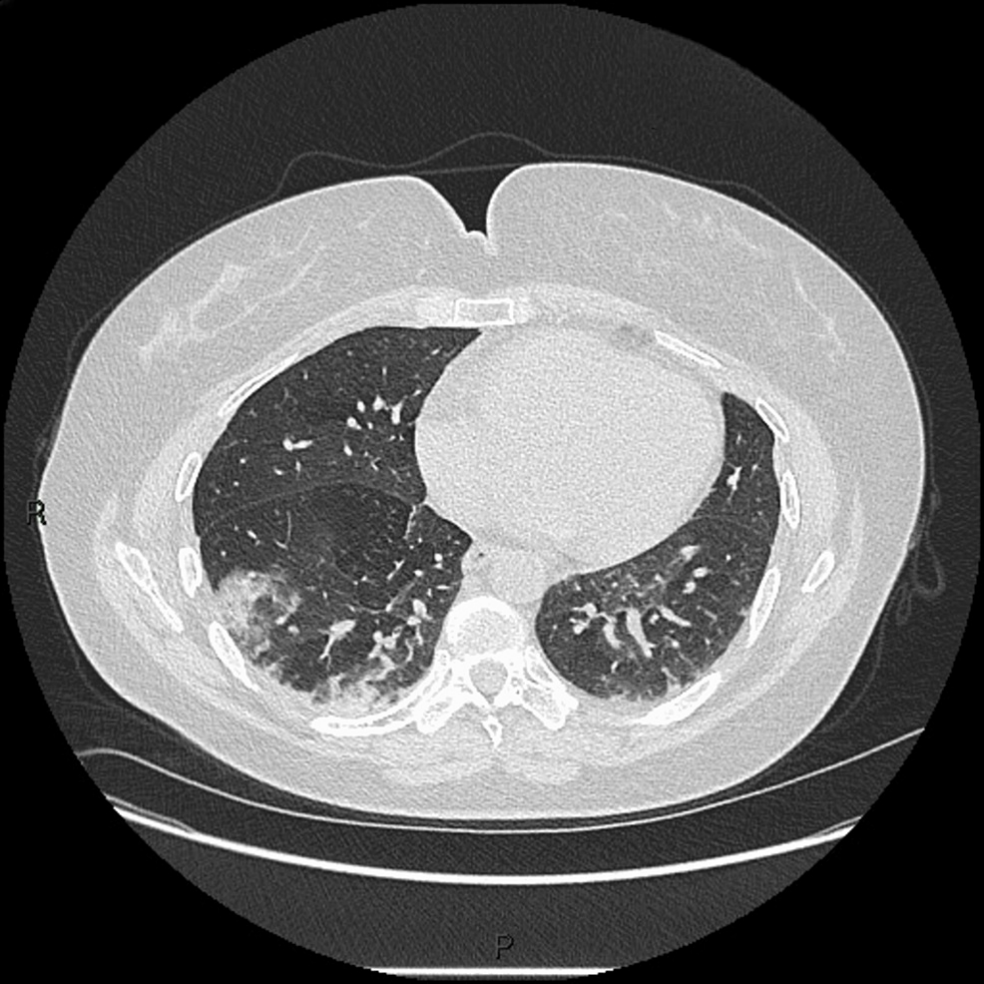
HRCT of patient 2 showing right middle lobe pneumonitis and ground-glass opacities HRCT: High-resolution computed tomography.

Ground-glass opacities were observed on the chest HRCT (Figure 8), and she was clinically diagnosed with pneumonitis. These investigations aided her definite diagnosis of anti-MDA5+ DM, and as the disease progressed the following year, she developed hair thinning, photosensitivity, mechanic’s hand, and arthralgia. A constant sensation of paraesthesia was observed in all fingers. Amyopathic dermatomyositis was diagnosed.

She was initially managed with 40 mg of oral prednisolone, 200 mg of hydroxychloroquine once daily, and naproxen. After a definite diagnosis, she was started on IVIG and mycophenolate, which was commenced at 500 mg twice daily (BD) and titrated up to 1 g BD. Her respiratory and joint symptoms improved; however, her mucocutaneous ulcers worsened with mycophenolate treatment. Finally, the patient was switched to prednisolone and rituximab. Antimicrobials were given for her recurrent lower respiratory tract infections (LRTIs) and infected Gottron’s papule. After two cycles of rituximab, her mobility, skin, and respiratory symptoms significantly improved.

## Discussion

One hypothesis for the pathogenesis of anti-MDA5+ DM involves environmental exposure in genetically susceptible individuals [7]. Evidence that this exposure may be due to a virus originates from epidemiological, geographical, and molecular studies. Epidemiologically, MDA5 DM has been shown to have a seasonal variation mimicking respiratory virus patterns, with increased incidence in fall and winter, a peak in late winter, and the lowest incidence in summer [8,9]. Geographically, it has been shown in Japan that there is an increased prevalence of MDA5 DM in rural areas and those living near freshwater compared to urban areas [10]. At the molecular level, MDA5 is an intracellular sensor of viral double-stranded ribonucleic acid (ds-RNA) [11,12]. It increases the production of type 1 interferons (IFN) and their downstream inflammatory mediators, which play an important role in the pathogenesis of anti-MDA5 DM [11,12]. Its expression is triggered by viral RNA.

The cases described in this article display the multifaceted nature of the clinical presentation of anti-MDA5+ DM. Despite this discrepancy, RP-ILD is a common feature of this subtype. This could be seen as widespread opacities on chest X-ray (CXR) or gound-glass opacity (GGO) on HRCT. Other radiographic findings included traction bronchiectasis and pneumomediastinum [5,6]. Pneumomediastinum was suggested as a prognostic marker in some case reports [5,6]. Several cases, including the two in this case series, portray the diagnostic and prognostic characteristics of elevated ferritin levels, as supported by the literature [4,13].

The individual treatment plans used in this study were tailored to the symptom profile of each patient. Apart from corticosteroids, a range of immunomodulators have been trialed. Mycophenolate treatment resulted in poor response in our patients. Azathioprine was effective in suppressing myositis and cyclophosphamide-controlled arthritis. One study showed that administering a combination of corticosteroids, tacrolimus, and cyclophosphamide as soon as possible gave better results than using a step-up regimen. Rituximab and IVIG generally improve disease control [3,8]. Aside from infected Gottron’s papules, vigilance should be maintained against respiratory infections. In one study, nearly 50% of MDA5+ DM patients tested positive for *Pneumocystis jirovecii* [9,11]. Of these cases, 20% had fatal outcomes [8,9]. Therefore, case 1 was kept on prophylaxis. A case report described the successful use of hemoperfusion with polymyxin B, followed by plasma exchange in a rapidly desaturating patient [14].

SARS-CoV-2 exposure, through vaccine or infection, can trigger the production of MDA5 and downstream mediators in the type 1 IFN pathway, even in healthy individuals [11,12]. A small study showed that 48.2% of all COVID-19 patients had increased MDA5 protein levels compared to healthy individuals [7,12]. Additionally, severe cases of COVID-19 are associated with impaired IFN-1 signaling, further suggesting a pathogenic link between anti-MDA5+ DM [11,13]. As of 2022, 19 cases of COVID-19-induced anti-MDA5+ DM have been reported [12,14]. Similar inflammatory cytokine profiles are seen in patients with COVID-19 infection and those with anti-MDA5+ DM [12,14]. It is hypothesized that cell damage caused by viral infection releases cytoplasmic MDA5 and increases the risk of autosensitization [11]. Although the patients included in this series initially had non-specific symptoms after being vaccinated, more symptoms characteristic of anti-MDA5+ DM began to develop faster after infection with the SARS-CoV-2 virus.

The benefit of including a few patients in this case series is that various methods were used to reach a definite diagnosis. This also allowed for the trial of different pharmacotherapy modalities and showcased their effects on disease progression. The patients and their relatives were very cooperative and willing to describe their experience with their treatment plans, giving a more holistic view of anti-MDA5+ DM. Additionally, the patients in this case series were jointly managed by the rheumatology, dermatology, pulmonology, and orthopedics departments, emphasizing the importance of a multidisciplinary approach. This study was limited by the small number of observed cases. There is also a limit to the timeline of disease progression, as the future disease course is yet to be determined.

## Conclusions

This series provides growing evidence of an intriguing association between COVID-19 exposure and anti-MDA5-positive dermatomyositis. Further research is required to elucidate the underlying pathogenic mechanism. Early involvement of a multidisciplinary team, consideration of symptom variety, infection vigilance, and impact on the quality of life are important factors for clinicians to consider when tailoring the management of these patients for optimized outcomes.

## Appendices

Patient views

Case 1

My symptoms first started after the first COVID-19 vaccine - I began feeling unwell with general weakness and fatigue. After my second vaccination, I developed a rash. These symptoms have significantly affected my job as a care assistant.

Once diagnosed, I was treated with several different medications. While this helped me, I also experienced significant side effects. Being on cyclophosphamide caused me to lose my hair, which was emotionally challenging. I was also told that I had developed osteoporosis and an increased risk of fractures. Additionally, I had a perforation in my esophagus, and most recently, I have gained a lot of weight, which I am told is a side effect of the steroids.

During the course of my treatment, I had a finger amputation, which significantly affected my daily life. I also lost my husband sometime before my diagnosis and had three children. Carrying on my life and rebuilding after my diagnosis have been emotionally challenging.

Looking back on everything I have been through, I am extremely grateful for all the care I have received. Despite my side effects, I am happy to remain here and I have a positive outlook on life.

Case 2

In December 2021, I received the Pfizer vaccine and, on January 17, 2022, I got the COVID. I had no symptoms and continued to train with weight and walking. One week later, I broke out with a rash on my face, which spread all over my body in different areas - a burning, itchy rash that was very painful.

By February, my hands and fingers swelled up, and I lost power in my fingers; I could not open a bottle or lift the kettle. I had no strength. I then developed shortness of breath and palpitations that lasted until July and August of 2023.

My symptoms over the last two years were as follows: Loss of hair; cuts and sores on my scalp; tinnitus; buzzing; blurred vision; shortness of breath; palpitations; anxiety; depression; loss of power in fingers, hands, and arms with very little strength; tremors, and internal shaking. Electric shock-type feeling through my whole body like I was wired, with small ulcers on my feet, hands, and ankles. Digital ulcers on my fingers that broke down into tendons. My toenails have crumbled, and I experienced circulation issues with my feet and hands, which usually turn purple and become painful. Sore hips and knees. Insomnia and a terrible fear of what was happening. My treatment has been steroids in high doses sometimes, rituximab to date three pulses, iloprost for a total of 10 days, and mycophenolate (2000 mg/day). This experience has been life-changing. I was a super fit, positive, healthy person with lots of energy, and it had been extremely challenging for myself and my family.
